# Update on Molecular Genetics of Gastrointestinal Stromal Tumors

**DOI:** 10.3390/diagnostics11020194

**Published:** 2021-01-28

**Authors:** Iva Brčić, Alexandra Argyropoulos, Bernadette Liegl-Atzwanger

**Affiliations:** Diagnostic and Research Institute of Pathology, Medical University of Graz, 8010 Graz, Austria; iva.brcic@medunigraz.at (I.B.); alexandra.argyropoulos@stud.medunigraz.at (A.A.)

**Keywords:** GIST, wild-type, KIT, PDGFRA, NF1, SDHB, SDH-competent, SDH-deficient

## Abstract

Gastrointestinal stromal tumors (GISTs) are the most common mesenchymal tumors of the gastrointestinal tract. The majority are sporadic, solitary tumors that harbor mutually exclusive *KIT* or *PDGFRA* gain-of-function mutations. The type of mutation in addition to risk stratification corresponds to the biological behavior of GIST and response to treatment. Up to 85% of pediatric GISTs and 10–15% of adult GISTs are devoid of these (*KIT/PDGFRA*) mutations and are referred to as wild-type GISTs (wt-GIST). It has been shown that these wt-GISTs are a heterogeneous tumor group with regard to their clinical behavior and molecular profile. Recent advances in molecular pathology helped to further sub-classify the so-called “wt-GISTs”. Based on their significant clinical and molecular heterogeneity, wt-GISTs are divided into a syndromic and a non-syndromic (sporadic) subgroup. Recently, the use of succinate dehydrogenase B (SDHB) by immunohistochemistry has been used to stratify GIST into an SDHB-retained and an SDHB-deficient group. In this review, we focus on GIST sub-classification based on clinicopathologic, and molecular findings and discuss the known and yet emerging prognostic and predictive genetic alterations. We also give insights into the limitations of targeted therapy and highlight the mechanisms of secondary resistance.

## 1. Introduction

Gastrointestinal stromal tumors (GISTs) are the most common mesenchymal neoplasms of the gastrointestinal tract with an annual incidence of approximately 10–15 cases per million [1,2]. They recapitulate the interstitial cells of Cajal (ICC) lineage/differentiation [3,4]. GISTs most frequently occur in the stomach followed by the small intestine (including duodenum) and rarely affect the colon/rectum and the esophagus. Metastases mainly occur in the liver and peritoneum [5]. They usually present sporadically in older adults (median age 60–65 years) with slight male predominance [6]. Up to 2% of GISTs occur in children with girls being more frequently affected. In addition, the association of GIST with various syndromes has been described, including neurofibromatosis-1 (NF1), familial GISTs, Carney triad (CT) and Carney Stratakis Syndrome (CSS) [1,7,8,9,10].

In 1998, Hirota et al. published a landmark paper demonstrating that activating mutations in the *KIT* gene, a transmembrane receptor with tyrosine kinase activity, is an oncogenic driver event in GISTs development [11] and in 2003, Heinrich et al. identified alternative mutations in the platelet-derived growth factor receptor alpha (*PDGFRA*) gene, as the second most common driver mutation in GISTs, showing that *KIT* and *PDGFRA* mutations are mutually exclusive [12]. Knowledge about the underlying genetic alterations revealed possible targeted treatment with tyrosine kinase inhibitors (TKIs) such as imatinib and sunitinib [13,14,15]. Continuous research efforts helped to further elucidate molecular insights of this disease and allowed the development of new treatment options based on the underlying molecular signature [14,16,17,18,19].

Nevertheless, the exact pathologic classification of GIST is the backbone of GIST treatment and is based on H&E (hematoxylin & eosin) morphology, immunohistochemistry (KIT, DOG1), risk stratification according to Miettinen and molecular testing at least in cases where treatment with TKI is planned [5,20].

**Figure 1:**
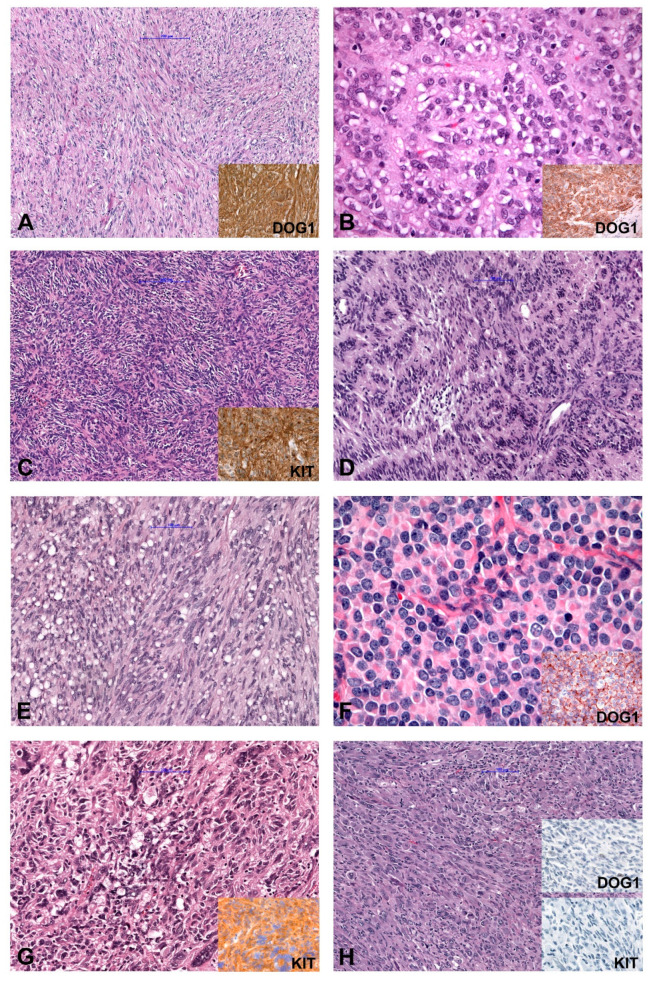
Morphology and immunohistochemical findings in GIST (Gastrointestinal Stroma Tumor). (**A**) Spindle cell GIST (inset: IHC DOG1+). (**B**) Epithelioid GIST (inset: IHC DOG1+). (**C**) Spindle cell GISTs with nuclear palisading, (**D**) GIST with storiform growth pattern, and (**E**) GIST with prominent paranuclear vacuolation. (**F**) Epithelioid GIST with a prominent plasmacytoid morphology. (**G**) Pleomorphic GIST (inset: IHC KIT+). (**H**) Dedifferentiated GIST (inset: IHC DOG1− and KIT−). IHC (immunohistochemistry); −(negative); +(positive).

In general, based on cytomorphology, three different morphologic patterns can be seen [2,21,22]. GISTs with spindle cell morphology (~70% of cases) are composed of cells with palely eosinophilic fibrillary cytoplasm, ovoid nuclei and ill-defined cell borders commonly with a syncytial appearance (Figure 1A). The cells are arranged in short fascicles or whorls. Epithelioid GISTs (up to 20% of cases) are composed of round cells with eosinophilic to clear cytoplasm arranged in sheets and nests (Figure 1B). GISTs with mixed morphology (~10%) are composed of both spindle and epithelioid cells. The cellularity is highly variable and collagenous, sclerotic or myxoid stromal changes can be seen in each subtype. The main differential diagnoses for all patterns are summarized in Table 1. Spindle cell GISTs can show nuclear palisading (Figure 1C), a storiform growth pattern (Figure 1D), and prominent paranuclear vacuolation (Figure 1E), a morphologic feature by far more commonly found in GISTs than in smooth muscle tumors. Epithelioid tumors can demonstrate a clear cytoplasm and a prominent plasmacytoid morphology (Figure 1F). The vast majority of GISTs are characterized by uniform and monotonous tumor cells. Nevertheless, pleomorphic GISTs and dedifferentiated GISTs are rarely seen (Figure 1G,H) [21,23,24,25,26,27].

In total, 95% of “classic” GIST express KIT by immunohistochemistry [28]. In addition, expression of CD34 in about 60–70%, smooth muscle actin (SMA) in 30–40%, S-100 protein in 5%, and desmin or keratin in 1–2% have been reported [22]. Moreover, discovered on GIST-1 (DOG1), a calcium-activated chloride channel protein has been shown useful to detect KIT negative tumors [29,30,31]. Specifically, the clone K9 showed the highest sensitivity and specificity for both KIT-positive and -negative tumors [32].

It is well known that approximately 80% of GISTs harbor activating mutations in the *KIT* or *PDGFRA* genes that are responsible for the up-regulation of crucial signaling pathways including MAPK and PI3K-AKT [2,12,33,34]. On the other hand, GISTs lacking *KIT* and *PDGFRA* mutations are referred to as “wild-type” (wt)-GISTs [35,36]. These tumors differ from *KIT* and *PDGFRA-*mutant GISTs with regard to their clinical behavior and heterogenetic molecular profile. Over the past few years, advances in molecular pathology helped to elucidate alternative molecular drivers in the non *KIT-* non *PDGFRA-* mutated so-called “wt”-GIST group. Alternative mutations, structural chromosomal and epigenetic changes have been demonstrated in this group making the molecular classification more complex. Recent insights about the crucial role of the SDH-complex, especially in the pathobiology of pediatric GISTs, helped to divide GISTs by immunohistochemistry in a succinate dehydrogenase B (SDHB)-retained and SDHB-deficient subgroup. This widely available screening approach can facilitate decisions on further molecular testing strategies.

This review will focus on the molecular genetics of classic *KIT/PDGFRA* mutated GIST and on the sub-classification of the wt-GIST group based on recent molecular findings. In addition, emerging prognostic and predictive genetic alterations will be discussed. Furthermore, insights into the limitations of targeted therapy and the mechanisms of secondary resistance will be highlighted.

## 2. Molecular Classification

**Figure 2:**
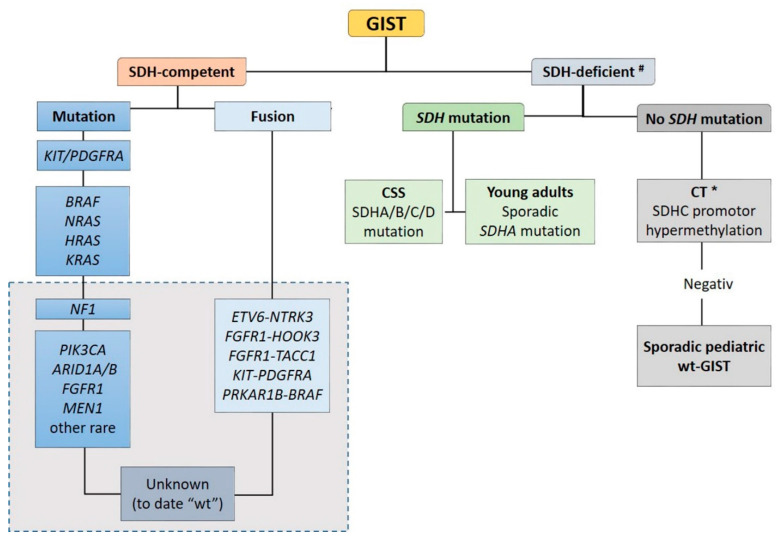
Sub-classification of GISTs into a succinate dehydrogenase (SDH)-competent and an SDH-deficient group by using an SDHB IHC ^#^. Legend: CSS: Carney–Stratakis Syndrome; CT: Carney triad; CT *: in some cases, mutations described [9]; wt: wild type. Grey rectangle: DNA- and RNA sequencing in a specialized center.

Recent advances in molecular pathology led to a better sub-classification of GISTs into an SDH-competent and an SDH-deficient group (by using an SDHB immunohistochemistry (IHC)), regardless of responsible mutation being acquired or inherited (Figure 2).

The SDH-competent tumor group includes: (i) *KIT-* and *PDGFRA-*mutated GISTs, (ii) GISTs with mutations in *BRAF*, *NF1, HRAS, NRAS,* and (iii) GISTs with exceedingly rare reported mutations in *ARID1A, ARID1B, CBL, FGFR1, ATR, LTK, SUFU, PARK2, ZNF217, KRAS, MEN1* and *PIK3CA*. Additionally, (iiii) GISTs harboring structural chromosomal changes such as *FGFR1-HOOK3*, *FGFR1-TACC1*, *ETV6–NTRK3, KIT-PDGFRA* and *PRKAR1B-BRAF* are placed within the group of the SDH-competent GIST [9,37,38,39,40,41].

The SDH-deficient tumor group includes wt-GISTs in association with CT, CSS or sporadic pediatric and so-called “young adult” GISTs [5,9,10,42].

The frequency of the most common genetic alterations found in GIST is presented in Table 2.

### 2.1. KIT/PDGFRA-Mutated GIST

#### 2.1.1. KIT-Mutated GISTs

**Figure 3:**
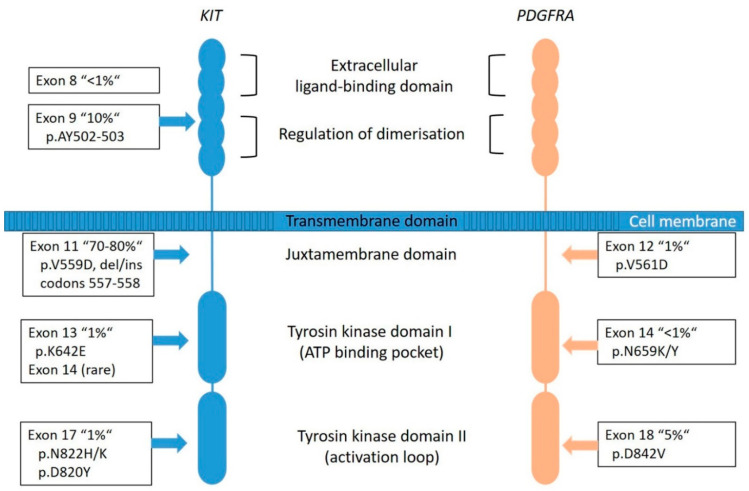
Graphical representation of *KIT* and *PDGFRA* transmembrane tyrosine kinase receptors with frequency and localization/distribution of primary mutations found in sporadic GIST (adapted from [53,54]).

*KIT* is a proto-oncogene and encodes the 145-kDa receptor tyrosine kinase (RTK) KIT, a transmembrane receptor with tyrosine kinase (TK) activity located on chromosome 4q11-q12 [5]. It is a member of the type III RTK family (together with PDGFRA, PDGFRB, the macrophage colony-stimulating-factor 1 receptor (CSF1R) and the receptor-type tyrosine-protein kinase FLT3 [43]. *KIT* consists of two main regions, the receptor regulatory domains (dimerization domain in the extracellular (EC) region, the transmembrane region and the juxta-membrane domain (JM) as well as the enzymatic domains the intracellular tyrosine kinase domains (TK[I] and TK[II]) (Figure 3) [44].

The RTK-KIT plays an important role in cell proliferation and differentiation (including Cajal cells) [3,45,46] and therefore plays a crucial role in the development of tumors (especially GIST, acute myeloid leukemia and melanoma) when it is mutated or upregulated [44]. These changes result in constant activation of the TK with consequent phosphorylation of substrate proteins without the presence of the corresponding ligand, called stem cell factor, influencing the intracellular signal transduction cascades, such as Ras/Raf/MAPK and PI3K/AKT pathways and leading to a constant autonomous activation causing an uncontrolled proliferation and inhibition of apoptosis [44,47,48].

Most of the *KIT* mutations in GISTs are somatic and are found in up to 80% of all tumors (Figure 2), rarely families with germline mutations have been described [8,34]. The mutations are found in different gene regions, including exons 8, 9, 11, 13, 14, 15, and 17. Mutations in exon 11 are the most frequent and are affecting the juxta-membrane domain (Figure 3). They are mostly caused by in-frame deletions within codon Gln550 and Glu560 (known as hot spot regions), missense-point-mutations mainly affecting codon Trp557, Val559, Val560 or Leu576 and duplications (especially in the 3′end) [21,49]. Deletions affecting codons 557–558 of exon 11 of the *KIT* gene have been reported in up to 28% of all GISTs and have been associated with high-risk tumors, having higher mitotic index (>5/50 HPF) and larger (>5 cm) tumor size [34]. Tumors showing this molecular profile occur equally in gastric and non- gastric location and arise in patients usually younger than 60 years. The local recurrence rate is lower compared to *KIT* exon 9, *PDGFRA* exon 18 and other *KIT* exon 11-mutated tumors. However, the prognostic power seems to be confirmed to the gastric location [34,50,51,52].

According to the Polish Registry, GIST with intron10/exon11 junction deletions (resulting in pK550_K558 deletion) and homo/hemizygous *KIT* exon 11 mutant GIST are rare tumors, accounting for 1.4% and 4% of all GISTs, respectively. Both are high-risk tumors with presumed aggressive/metastatic behavior and early metastatic disease/disseminated disease at presentation, respectively [34]. In contrast, single nucleotide substitutions and duplications (exclusively gastric location) are associated with benign clinical outcome [34,51]. Similar findings were very recently published by Shen et al. [53].

The mutations in exon 9 are found in approximately 10% of cases and are interfering with the extracellular parts (duplications of Ala502- Tyr503) (Figure 3). These tumors are commonly located in the small bowel and are often associated with a more aggressive phenotype [49]. Primary KIT mutations can also occur in exon 13 (TK[I]: ATP binding pocket) and exon 17 (TK[II]: kinase activation loop), but these mutations are rare (~2%) and data are quite limited [55]. *KIT* exon 13 and exon 17 mutant GISTs are more frequently found in the small bowel, usually have spindle cell morphology and most of them have the same behavior when compared to other GISTs. Gastric *KIT* exon 13 mutant is an exception as they tend to be slightly larger and more aggressive than gastric GISTs on average [55,56].

#### 2.1.2. PDGFRA-Mutated GISTs

*PDGFRA* is a typical RTK and is, as well as *KIT*, located on chromosome 4q11-q12. Together with its ligand platelet-derived growth factor (PDGF), it is responsible for many physiological processes of growing and development in the human body. The receptor is similarly constructed as KIT (Figure 3). The PDGFs are divided into five isoforms: *PDGF-AA*, *PDGF-AB, PDGF-BB*, *PDG-FCC* and *PDGF-DD*, and bind to the receptors *PDGFRA* and *PDGFRB* [57]. Particularly, the *PDGFRA* is important for lung, skin, intestine, skeleton, gonads and is an essential factor in embryonic development. Normally, after binding to the receptor, phosphorylation activates signal cascades (Ras/Raf/MAPK and PI3K) [57]. Due to genetic aberrations, the *PDGF* signal is unrestrained active in neoplastic cells, which leads to ligand-independent phosphorylation and therefore uninhibited proliferation. Additionally, it plays a role in the epithelial-mesenchymal-transformation. *PDGFRA-*mutated GIST account for approximately 8–10% of GISTs (Figure 2); however, their lower representation in clinical trials can be explained by a comparatively benign clinical behavior of these tumors.

Most frequently, mutations are localized in the exon 18 (Figure 3) that codes for the activation loop in the TK domain and represents about 80% of the *PDGFRA-*mutated GISTs [58]. Mostly, these are missense mutations that result in the substitution of Asp to Val in codon 842 (D842V), a mutation known to be imatinib-resistant [16,51]. Further mutations are described in exon 14 and rarely in exon 12. Exon 14 correlates to the TK domain and exon 12 to the juxta-membrane. Typically, *PDGFRA-*mutated GISTs show an epithelioid pattern and are located in the stomach [12,29,31]. *PDGFRA* exon 18 mutation status correlates with an extremely favorable disease outcome compared to *KIT* exon 9 mutations and *KIT* deletions involving codons 557 and/or 558 of exon 11 [50]. Nevertheless, cases in the stomach that progressed (11 of 14 cases) carried an exon 18 *PDGFRA* D842V substitution [50].

#### 2.1.3. GIST Genomic Progression Model

In most GISTs (including micro GISTs), *KIT*, *PDGFRA*, *NF1 or SDH* mutations are the initiating oncogenic drivers. However, additional stepwise accumulation of chromosomal aberrations is necessary/essential for further tumor progression. The earliest aberration found in up to 70% of cases is the loss of 14q [59]. Recently, somatic homozygous inactivating mutations of the chromosome 14q of the *MYC-associated factor X (MAX)* gene has been identified as a common early step in the progression of GISTs (microGISTs and low-risk GISTs) [60,61]. The inactivation of the MAX tumor suppressor leads to a p16 inactivation and an increase of proliferation in early tumors. In intermediate and high-risk GISTs further alterations have been described, namely, losses of 22q, 1p, 15q, 13q and 9p (spanning CDKN2A or p16INK4A) and/or gains at 5q, 8q, 16q and 20q [62,63,64,65,66]. These cell cycle dysregulating events result in inactivating mutations in other tumor suppressors such as *p16*, *RB1*, *TP53* and cause the transition to high-grade GISTs [67,68,69]. Furthermore, it has been demonstrated that inactivation of dystrophin, encoded by the *DMD* gene on Xp21.1, contributes to permissiveness for metastatic behavior in GIST and was found in approximately 90% of metastatic GISTs [70].

#### 2.1.4. Resistance Mechanisms in GIST

The vast majority of patients with unresectable or metastatic GIST respond to imatinib treatment. Treatment response can be demonstrated on CT scan as a reduction of the tumor mass or as decreased FDG uptake on a PET scan.

Resistance to imatinib, primary and secondary, can be partially explained by a conformational shift in the kinase domain of KIT and PDGFRA that favor the activated state [71]. Imatinib can only bind to the inactive conformation of both the KIT and PDGFRA receptors. For example, the *PDGFRA* D842V mutation, a known imatinib resistance mutation, results in a distortion of the kinase activation loop, thus strongly tilting the protein conformation in favor of the activated structure.

Tumor progression within the first 6 month of treatment is known as primary resistance. In this group patients with GISTs harboring a *PDGFRA D842V* mutation are therefore over-represented in the primary resistant GIST group as well as wt-GIST and *KIT* exon 9-mutated GISTs initially treated with only 400 mg of imatinib [16,51].

**Figure 4:**
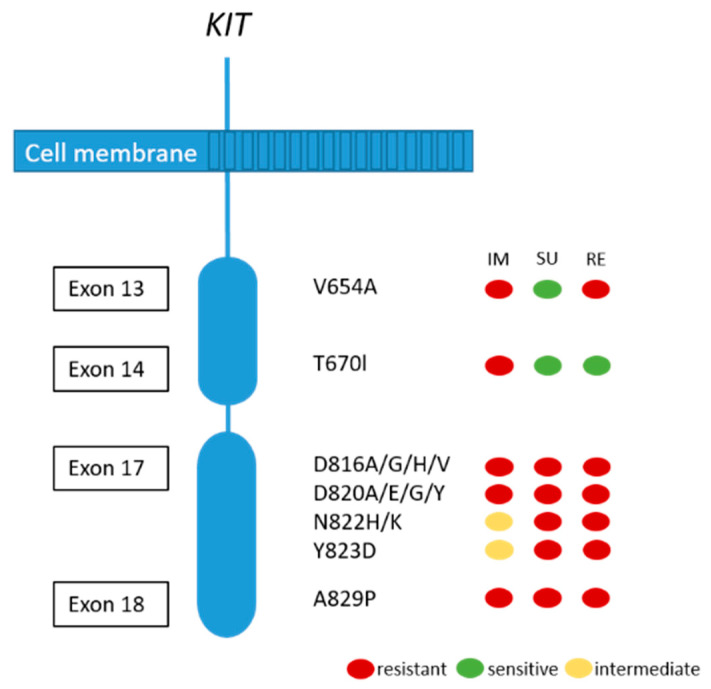
Secondary KIT mutations and predictive response to most frequently used TKIs (IM:imatinib; SU: sunitinib; RE: regorafenib). Adapted from [80].

Although the majority of patients show a good response or stable disease under imatinib treatment, tumor progression in one or more lesions usually occur after 12–36 months. This finding is called secondary resistance and is most frequently caused by secondary acquired mutations in the KIT kinase domain. Rarely, other resistance mechanisms including *KIT/PDGFRA* genomic amplification and activation of alternative oncogenes have been reported [51,72]. Secondary *KIT* kinase mutations are non-randomly distributed single nucleotide substitutions affecting codons in the ATP binding pocket (exons 13 and 14) and the kinase activation loop (exon 17 and 18) (see Figure 4) [72]. Acquired secondary resistance mutations are described in up to two-thirds of GISTs progressing after treatment with tyrosine kinase inhibitors TKI [73,74,75,76]. A very common secondary resistance mutation is the *V654A* in exon 13 of the *KIT* gene [72,77]. Secondary mutations are found to be significantly more common in GISTs with primary *KIT* exon 11 mutations than in those with exon 9 mutations. In tumors with primary *PDGFRA* mutations, secondary mutations in exon 18 with a primary mutation in exon 12 have been rarely described [77]. While sunitinib has been shown to be effective against secondary mutations located in the ATP binding pocket (exon 13 and 14), this drug is not effective against mutations in the kinase activation loop (exon 17 and 18) based on in vitro and in vivo studies [14,77]. Recently, Zhang et al. have demonstrated that cabozantinib, in a mouse model with a V654A second site *KIT* mutation, might be a more effective drug in overcoming secondary resistance than sunitinib. In addition, they concluded that second-side mutations are not only responsible for drug resistance, but also for changing the oncogenic potential and activation of different signaling pathways, in this case, KIT-dependent STAT activation [78]. Unfortunately, knowledge about the substantial inter- and intralesional heterogeneity of TKI resistance mutations in metastases of patients treated with imatinib alone or imatinib and sunitinib challenges the potential treatment options and tissue selection for mutational analysis [72]. Therefore, the use of liquid biopsy has been shown to be feasible to search for resistance mutations [79].

Recently, a new treatment option with a potent KIT/PDGFRA inhibitor avapritinib, with substantial clinical activity in patients with the *PDGFRA* D842V mutation, became available [17]. However, secondary resistance against avapritinib can occur and is caused by secondary *PDGFRA* mutations in exons 13, 14 and 15 (PDGFRA kinase domain) that interfere with the avapritinib binding site [81].

#### 2.1.5. Prognosis and Mutational Status in Treatment-Naïve GIST

The prognostic value of mutational status was nicely demonstrated in the study analyzing a series of 451 untreated primary localized GIST for *KIT*, *PDGFRA* and *BRAF* mutations finding that the mutational status is a significant prognostic indicator of overall survival (OS) [6]. Based on multivariable Cox regression models, the authors identified three distinct molecular risk groups. Group I, consisting of *PDGFRA* exon 12, *BRAF* and *KIT* exon 13-mutated cases, exhibited the best clinical outcome. Group II, the intermediate-risk group, included *KIT* exon 17, *PDGFRA* exon 18 D842V and *PDGFRA* exon 14-mutated GISTs. Group III, displayed the worst clinical outcome and was comprised of *KIT* exon 9 and exon 11 and *PDGFRA* non-D842V exon 18 mutant GISTs [6]. This study highlights the prognostic impact of the mutational status in the natural history of GIST. Therefore, the inclusion of molecular data together with risk stratification criteria can clearly help to enhance the decision-making process, especially in the adjuvant setting.

#### 2.1.6. Genetic Subtypes of GIST—Impact on Treatment Response

In the last two decades, a growing body of evidence showed that the mutational status in GIST is a strong predictive indicator of response to treatment. Imatinib mesylate (STI571, Gleevec™, Novartis Pharmaceuticals, Basel, Switzerland) is an oral selective inhibitor of a number of TK including *KIT*, *PDGFR*, *ABL* and *BCR-ABL*. Since 2000, it has been used in GIST therapy in patients with metastatic/advanced disease and has become a paradigm in the treatment of solid tumors with targeted therapy, tremendously changing the survival of these patients [82]. Subsequently, other TKI, namely, sunitinib, regorafenib, and very recently ripretinib and avapritinib, have been approved and are mainly used in an advanced (recurrent and metastatic) disease after ineffective imatinib treatment or in the context of selective mutations like *PDGFRA* D842V [17,18,20,83].

Studies demonstrated the prognostic significance and prediction to treatment response in certain types of mutations detected in GIST (see Table 1). Tumors with common *KIT* exon 11 mutations, at codon 557/558, especially if located in the stomach, are associated with more aggressive behavior, higher risk of disease recurrence and increased risk of developing metastases [50,51,84]. Therefore, including this molecular information especially in the decision process if a neoadjuvant therapy showed be given seems to be of relevance. Tumors with a mutation in *KIT* exon 9 were shown to be imatinib sensitive; however, these GIST require a double dose (800 mg/daily) [14]. In *PDGFRA-*mutated tumors, substitutions involving codon *D842* in exon 18 (including D842V, RD841-842KI, DI842-843IM) are primarily resistant to both, imatinib and sunitinib [12,14,77]. In contrast, further mutations in exon 18, including D842Y, D846Y, N848K, Y849K, HSN845-848P and mutations in exon 14 of the *PDGFRA* gene are sensitive to imatinib [12]. However, very recently avapritinib has been approved for the treatment of advanced *PDGFRA D842V*-mutant GIST [17].

In the pediatric GIST group, disease progression has been shown to occur later if they are on sunitinib therapy than on imatinib, leading to the conclusion that these patients benefit more from sunitinib therapy in the first line of treatment [15,85].

Taking all this into account, the European Society for Medical Oncology (ESMO) recommend routine analysis of GIST associated mutations for better planning of adjuvant therapy, with the possible exclusion of <2 cm non-rectal GISTs [20].

#### 2.1.7. Morphological Changes after TKI Therapy

Treatment with TKI can influence GIST morphology [23,27]. A dramatic decrease in tumor cellularity as well as prominent stromal alterations including marked sclerosis and myxoid change can occur. However, in the vast majority of cases, the cytomorphology remains comparable with the primary tumor. Nevertheless, marked changes in the tumor morphology have been described. Very rare but well described is the so-called dedifferentiated GIST, frequently showing an abrupt transition from a “classic” GIST morphology in a dedifferentiated component.

Dedifferentiation in GIST is commonly associated with long term TKI treatment but can also occur de novo [23,24,25,27]. The dedifferentiated component shows an anaplastic/pleomorphic appearance, high nuclear atypia, high mitotic activity, and necrosis. Various histologic patterns have been reported in this context including rhabdomyosarcoma [25,86], angiosarcoma [23], or undifferentiated pleomorphic and spindle sarcoma. Usually, dedifferentiation is not associated with additional mutations in the original driver oncogene. Instead, these tumors show genetic instability, indicated by LOH or low-level *KIT* amplification [23]. Therefore, the dedifferentiation process might be caused by the activation of alternative pathways driven by *KIT*-independent oncogenic mechanisms. Nevertheless, the possibility of dedifferentiation in GISTs should always be considered when an undifferentiated sarcoma component is seen in the gastrointestinal tract. In this context, extensive sampling of tumor tissue, patients’ clinical history and molecular analysis are diagnostically helpful.

#### 2.1.8. Familial GIST

Familial GIST syndrome is defined by germline mutation of *KIT* or *PDGFRA*, the occurrence of multiple GISTs, hyperpigmentation, mast cell tumors and dysphagia due to ICC hyperplasia [87,88]. To date, approximately 50 cases have been described. The most common *KIT* and *PDGFRA* mutations observed in individuals with familial GIST are summarized in Table 3 [8,87,88,89,90,91,92,93,94,95,96,97,98,99,100,101].

The vast majority of the patients develop multiple GISTs by middle age. The morphology of tumors in this setting is similar to sporadic GISTs and they are caused by a monoclonal proliferation of tumor cells. Additionally, diffuse proliferation of the Cajal cell population is found causing ICC hyperplasia, representing, however, a non-neoplastic polyclonal nature [88,102].

### 2.2. Wild-Type GIST

**Figure 5:**
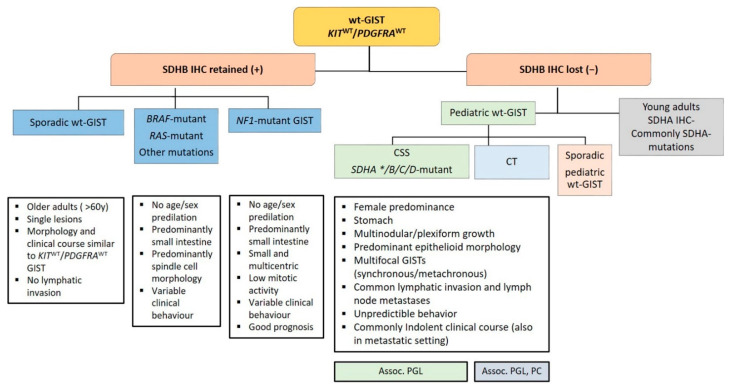
Diagnostic algorithm in wild-type gastrointestinal stromal tumors (wt-GISTs). Legend: Assoc.: associated; CSS: Carney–Stratakis Syndrome; CT: Carney triad; IHC: immunohistochemistry; PC: pulmonary chondroma; PGL: paraganglioma; SDHB: Succinate dehydrogenase B; wt: wild type; *: rarely reported.

Approximately 10–12% of all GISTs lack mutations in *KIT* and *PDGFRA* and are called wt-GISTs. Over the last few years, it became apparent that this group is heterogeneous with regards to clinical phenotype and molecular characteristics [103]. Based on recent advances in molecular pathology, wt-GISTs can be sub-classified in an SDH-competent and an SDH-deficient group, irrespective of whether they are sporadic or familial/genetic [9]. Immunohistochemical screening for SDH deficiency became a powerful and convenient screening tool to stratify GIST patients (especially pediatric and young adult patients with GIST located in the stomach) into these two groups (see Figure 5).

**Figure 6:**
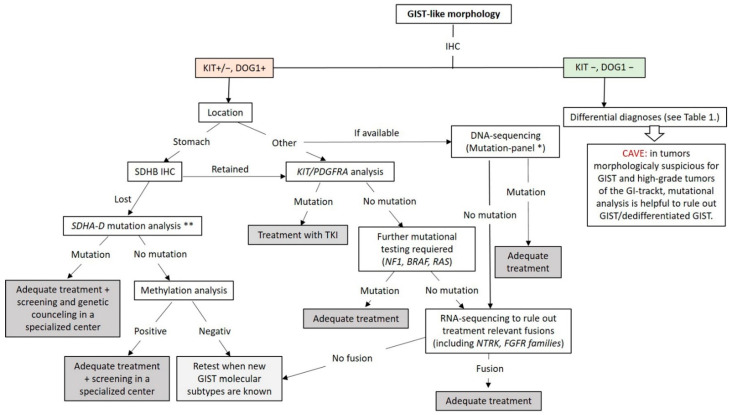
Diagnostic and molecular testing algorithm for GIST. * Our GIST mutation-panel includes following genes: PDGFA, KRAS, NRAS, HRAS, BRAF, KIT (Exon 8,9,10,11,12,13,17,18), PDGFRB (Exon 12,13,14,17,18), TP53 (Exons 4–10), SDHA, SDHB, SDHC, SDHD, NF1, CDKN2A and RB1. ** If available, mutation-panel is performed. TKI: Tyrosin Kinase Inhibitor.

At this point, genetic testing using DNA- and RNA-NGS can be used to exclude the known genetic changes (mutations and fusions) in GIST. The workflow applicable in routine practice is shown in Figure 6. GISTs lacking these known molecular changes should be collected for research in specialized centers.

#### 2.2.1. SDH-Competent wt-GISTs

##### NF1-Mutant GIST

NF1 is an inherited, autosomal dominant disease phenotypically characterized by multiple café-au-lait spots, Lisch nodules, freckling neurofibromas, and occasional development of malignant peripheral nerve sheath tumors. Approximately 7% of patients with NF1 develop GIST during their lifespan [7]. NF1-associated GISTs are commonly multicentric (with or without multinodular growth pattern), predominantly located in the small intestine and lack *KIT* and *PDGFRA* mutations [104,105]. These tumors express KIT, DOG1 and SDHB by IHC [106]. NF1-associated GISTs frequently demonstrate loss of heterozygosity at 14q and 22q similar to sporadic *KIT*- and *PDGFRA*-mutated GIST [107]. In addition, it has been suggested that somatic inactivating *NF1* mutations outside the context of NF1 may be the oncogenic mechanism for a subset of sporadic adult wt-GIST [60,104,105]. Moreover, an NF1- mutant GISTs may harbor the additional cancer-related mutations, like inactivating Notch pathway mutations including *NOTCH2*, *MAML2* and *CDC73,* most frequently found in tumors at the duodenal-jejunal flexure (ligament of Treitz) [105].

##### BRAF, KRAS and PIK3CA-Mutant GISTs

*BRAF* mutations (V600E) have been found in approximately 8–13% of wt-GISTs, and to date, *BRAF* and *KIT/ PDGFRA* mutations seem to be mutually exclusive [35,41,108,109]. According to available data, pediatric and young adult wt-GISTs are practically devoid of *BRAF* mutations, with only a single pediatric wt-GIST reported as *BRAF-*mutated so far [110]. *BRAF*-mutant GISTs equally affect men and women, are commonly associated with small bowel manifestation amd show spindle cell morphology and variable clinical behavior. Currently, no clinical or prognostic correlations have been linked to *BRAF* mutation status [111].

*KRAS*-mutated GISTs are exceedingly rare and the clinicopathologic features are not fully elucidated yet [112].

*PIK3CA* mutant GISTs are also exceedingly rare. In the largest study to date 10 (8 primary and 2 metastatic GISTs) out of 529 imatinib-naïve GISTs demonstrated PIK3CA mutations. *PIK3CA* mutations were associated with large tumor size and aggressive clinical behavior [113]. As the number of reported patients is small, further studies with good follow up is required.

##### GISTs with ETV6-NTRK3-Fusion

**Figure 7:**
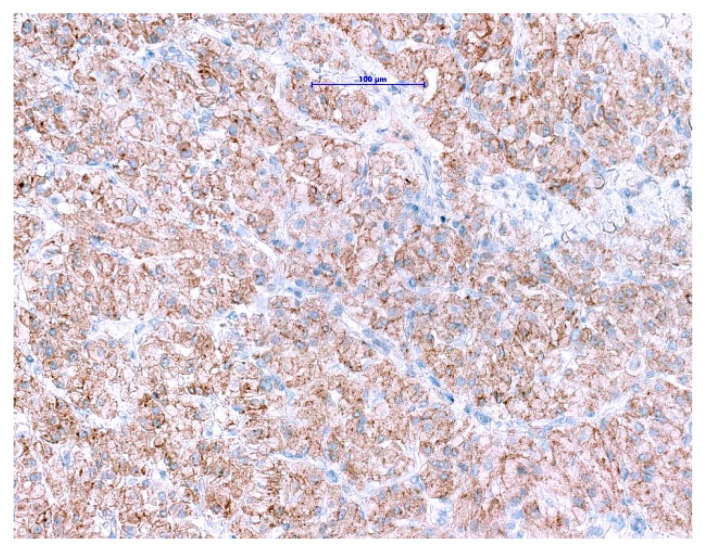
Immunohistochemical staining with pan-TRK antibody in GIST shows diffuse cytoplasmic and membranous expression. (Scale bar shows 0.1 mm).

*NTRK* gene fusions appear to be primary oncogenic drivers in *NTRK*-rearranged tumors. Although GISTs with *ETV6-NTRK3* fusions are exceedingly rare, approved highly potent NTRK-inhibitors are now available. The excellent clinical response and outcome in patients harboring *NTRK*-rearranged tumors treated with these agents have been demonstrated in several studies [114,115,116]. Even though pan-TRK IHC is wildly used as a reliable and affordable screening method for the detection of *NTRK*-fusions in most of the pathology departments [117,118], it has its limitations. The positive expression has been reported in a subset of neoplasms with neuronal and smooth muscle differentiation, as well as GIST, where diffuse, moderate to strong cytoplasmic pan-TRK expression has been described (Figure 7) with lack of an *NTRK1-3*-fusions by RNA sequencing [119]. Therefore, fusion analysis should be performed in SDHB-retained and mutation-negative cases, to enable optimal patients management especially in the metastatic setting.

#### 2.2.2. SDH-Deficient wt-GISTs

A unique group within the wt-GISTs are the SDH-deficient GISTs. The key oncogenetic mechanism of these tumors is an energy metabolism defect in the SDH complex (mitochondrial complex or succinate reductase) which is composed of 4 subunits SDHA, SDHB, SDHC and SDHD, mapping to 5p15.33, 1p36.13, 1q23.3, and 11q23.1, respectively. The SDH enzyme is a key enzyme in the Krebs cycle and electron transport chain. It is a highly conserved heterotetrameric protein that consists of SDHA and SDHB being part of the catalytic unit and SDHC and SDHD are membrane-anchoring subunits. Genetic or epigenetic alterations in any of the subunits lead to accumulation of succinate which is a competitive inhibitor of α-ketoglutarate- dependent dioxygenases, including the TET family of 5-methylcytosine hydroxylases [120]. Members of the TET family are active DNA demethylases that convert 5-methylcytosine to 5-hydroxymethylcytosine, and inhibition of TET activities can lead to aberrant DNA methylation in GISTs. A genome-wide DNA methylation analysis of SDH-deficient GISTs revealed greater DNA hypermethylation than in GISTs with *KIT* mutation [121]. Activated cellular pathways are leading to increased angiogenesis and cellular proliferation are activated [122]. Accumulation of succinate causes stabilization of HIF1-α, which controls oncogene transcription [123].

Insulin-like growth factor 1 receptor (IGF1R) is overexpressed in *KIT*/*PDGFR* wt-GISTs and is particularly elevated in SDH-deficient GISTs [124,125,126]. The IGF family is composed of two ligands (IGF1 and IGF2), two receptors (IGFR1 and IGFR1) and 6 IGF binding proteins (IGFBPs). Activation of IGFR results in activation of downstream signals, including the MAPK and PI3K/AKT pathways [127]. Inhibition of IGF1R induces apoptosis and represses AKT and MAPK signaling in GIST cells, which implicates the IGF signal in the development of SDH-deficient GISTs [128]. Very recently, it has been shown that two RTK genes (*KIT* and FGF receptor 1 (*FGFR1*)), as well as *FGF3* and *FGF4* oncogenes are most highly expressed in SDH-deficient tumors [129]. These findings may explain the poor response of TKIs in SDH-deficient GISTs.

Alterations of a single SDH subunit can be reliably detectable by loss of SDHB expression using IHC. Therefore, SDHB IHC can be used as a convenient tissue-based screening method for genetic and epigenetic alterations in the SDH complex [103].

**Figure 8:**
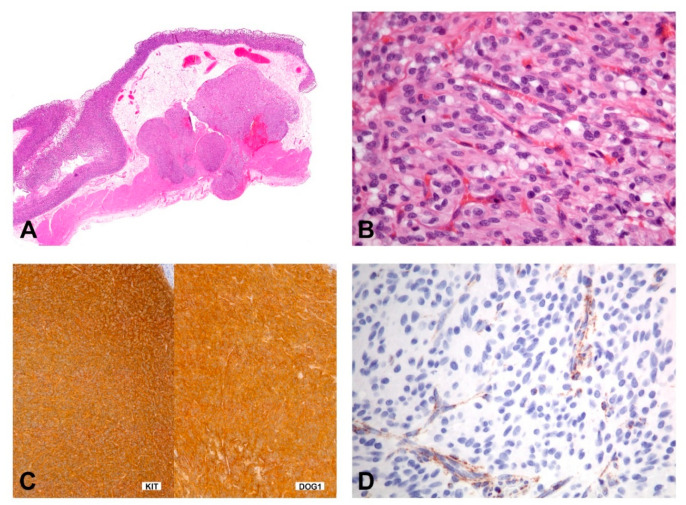
Morphology and immunohistochemical findings in SDHB-deficient GIST. (**A**) The gastric tumor with multilobulated/plexiform growth pattern. (**B**) The tumor is composed of epithelioid cells with a syncytial appearance. (**C**,**D**) Immunohistochemically, the tumor shows positivity for KIT and DOG1 (**C**), while the expression of SDHB is lost (**D**); the cytoplasmic stain of the endothelial cells shows the positive internal control).

SDH-deficient GISTs usually occur in patients younger than 40 years of age, have a female predilection, occur in the stomach (most commonly antrum) and have a spectrum of behavior from indolent to progressive. The tumors show characteristic morphologic features including a multinodular growth pattern (Figure 8A), the occurrence of multiple tumors, lymphovascular involvement and lymph node metastasis. Morphologically, these tumors are epithelioid or mixed epithelioid/spindled (Figure 8B). Mitotic activity may reach more than 5 per 5mm ^2^; however, risk stratification, according to Miettinen, is not working in this context [130]. Tumors consistently express KIT and DOG1 by IHC (Figure 8C) and show loss of SDHB staining (Figure 8D). However, the mechanism of *KIT* activation in SDH-deficient GISTs remains unclear. They lack the canonical chromosomal alterations observed in *KIT/PDGFRA/NF1*-mutant GISTs (i.e., loss of 14q, 22q, 1p, and 15q) and, instead, may show 1q deletion, presumably involving the SDHC locus [131,132].

#### 2.2.3. Syndromic SDH-Deficient wt-GIST

##### Carney Triad (CT)

CT is a very rare disease with the synchronous or metachronous occurrence of at least three different tumor entities; GIST, paraganglioma (PGL), and pulmonary chondroma. CT is never inherited and affects mostly females. Most cases of CT show down-regulation of SDH through site-specific hypermethylation of the SDHC gene [133,134]. In most cases, the *SDHx* epigenetic downregulation leads to downstream activation of the HIF signaling pathway. Overexpression of insulin-like growth factor receptor type 1 (IGFR1) at the protein level has been found in the majority of SDH deficient GIST; however, the exact molecular mechanism for this overexpression is currently not known.

Killian et al. studied SDH deactivation through genome-wide DNA methylation and expression study. This study included 59 SDH-deficient GIST and showed that 94% of tumors lacking SDH mutations showed *SDHC* promoter-specific CpG island hypermethylation and subsequent gene silencing. This fact led to the hypothesis that *SDHC* epimutation could be the main molecular mechanism that leads to succinate dehydrogenase enzyme dysfunction in SDH-deficient GIST that lack SDH mutations [121]. In 2016, Boikos et al. reported that 84/95 wt-GISTs lacked SDHB expression by IHC. Molecular analysis of the SDHB deficient GISTs revealed that 2/3 of the cases demonstrated mutations in SDH subunits whereas 1/3 showed *SDHC* promoter methylation. Mutations in SDH subunits were associated with CSS whereas *SDHC* promoter methylation was the main molecular characteristic of GIST in CT [9]. Only a few cases of CT are reported to have SDH subunit mutations, suggesting a partial overlap between the two conditions. Nevertheless, both lead to increased methylation of the entire genome in these tumors [9].

##### Carney-Stratakis Syndrome (CSS)

CSS is characterized by gastric multifocal GISTs and PGL. CSS shows an autosomal-dominant, with incomplete penetrance, inheritance pattern affecting both genders during childhood and adolescence. SDH deficiency is caused by inactivating germline mutations or large deletions in the *SDHB*, *SDHC* or *SDHD* (rarely *SDHA*) genes encoding the subunits B, C or D of the SDH enzyme [10,135,136].

In CSS, in contrast to CT, DNA methylation patterns were identified only at a few of the CpGs located close to the SDHB gene [133]. In these patients, the SDHC gene promoter was completely unmethylated in all screened CpG sites supporting the hypothesis that the CSS is a truly different entity from CT.

##### SDHA-Deficient wt-GIST

Approximately 30% of SDH-deficient GISTs demonstrate loss of expression for SDHB and SDHA by IHC. Loss of SDHA expression is a strong indicator of mutations in the *SDHA* gene. Most of the cases demonstrate germline mutations. The most common *SDHA* mutation detected in these patients is the c.91C4T; p.R31X. Simultaneous allelic loss at the SDHA locus at 5p15 has been detected with comparative genomic hybridization. Mutations in this tumor suppressor follow a classic 2-hit hypothesis. Loss of SDHA protein expression is associated with both truncating and missense germline mutations. *SDHA* mutation associated GISTs occur at an older age than other SDH-deficient GISTs, with the median age of presentation in the largest series being 34 years [42,135].

#### 2.2.4. Treatment Options in GISTs without Currently Druggable Target

Surgical management is considered the main treatment option for non-metastatic wt-GIST and it should also be considered as a treatment option in the metastatic setting, in the so-called wt-GIST group, if a druggable target (see molecular screening approach) cannot be detected [137]. Systemic treatment in metastatic wt-GIST showed no objective tumor response to imatinib, but a superior response to sunitinib, especially in the pediatric GIST group [138].

The SDH-deficient GIST group is mainly composed of pediatric GIST patients including patients with CSS and CT, whereas only a small subset of sporadic adult (especially young adult) patients fall into this group. The underlining genetics for loss of the SDHB expression by IHC is heterogeneous including somatic mutations and germline mutations in *SDHA/B/C/D* as well as promotor hypermethylation or deletions [9,42,133].

## 3. Conclusions

In the last two decades, molecular pathology has massively improved our understanding of GIST development. Identification of targetable genetic alterations has subsequently changed treatment approaches and brought survival benefits for the vast majority of GIST patients. Mutational analyses have been shown to have a prognostic and therapeutic impact. Understanding of drug resistance mechanisms contributed to the development of novel therapeutic strategies/targets. Avapritinib is one example of a new generation TKI that greatly improved treatment, especially for patients with a *PDGFRA D842V* mutation. Nowadays, comprehensive NGS based molecular testing strategies can facilitate to detect clinically relevant targets, including *NTRK* fusions. Therefore, a comprehensive molecular workup in a specialized center is needed if the common *KIT/PDGFRA* mutations in a GIST cannot be detected.

## Figures and Tables

**Table 1:** Summary of the most frequent differential diagnosis for spindle cell, epithelioid and mixed GIST, including immunohistochemistry and molecular findings.

Morphology	KIT/DOG1 IHC	Diagnosis	Additional Positive IHC	Helpful Genetic Alteration(s)
**Spindle cell**	KIT+, DOG1+	GIST	CD34 (70%), SMA (30%)	*KIT, PDGFRA* and others
	KIT− (or very weak), DOG1-	Leiomyoma /Leiomyosarcoma	Desmin, SMA, caldesmon	
		Schwanomma	S100, SOX10 (nuclear)	
		Solitary fibrous tumor	CD34, STAT6 (nuclear)	*NAB2-STAT6*
		Fibromatosis	beta-catenin (nuclear)	*CTNNB1* or *APC* mutation
		IMT	ALK	*ALK* and *ROS1 (rare)* rearrangements
		DDLPS	MDM2, CDK4	MDM2, CDK4 amplification by FISH
		Inflammatory fibroid polyp	CD34	*PDGFRA mutations*
**Epithelioid/mixed**	KIT+, DOG1+	GIST	SDHB retained/deficient (stomach)	*KIT, PDGFRA, SDHA-D,* *SDHC* promotor hypermethylation
	KIT+/−, DOG1+/−	GIST		*PDGFRA*, *KIT* mutations
	KIT−, DOG1−	PEComa	SMA, HMB45, MelanA, Desmin, TFE3 (subset of cases)	*TSC2* mutation, *TFE3*-fusions
		Melanoma metastasis (can be KIT+)	SOX10, S100, HMB45, Melan A	cave: the common *BRAFV600E* mutation can be also found in GIST
		Glomus tumor	SMA, caldesmon	*NOTCH* rearrangements and/or *BRAF mutations (p.Val600Glu)*
		Neuroendocrine neoplasms	cytokeratin, synaptophysin, chromogranin A	*DAXX, ATRX, p53, RB1 mutations*

Legend: ALK: anaplastic lymphoma kinase; DDLPS: dedifferentiated liposarcoma; FISH: fluorescence in situ hybridization; GIST: gastrointestinal stromal tumor; IMT: inflammatory myofibroblastic tumor; PEComa: perivascular epithelioid tumor; SDHB: succinate dehydrogenase B; SMA: smooth muscle aktin.

**Table 2:** Anatomic distribution, frequency and treatment response of the most common GISTs groups.

Genetic Type		Frequency	Anatomic Location	Treatment
***KIT* mutations**				
Exon 8		<0.1%		
Exon 9		6%	small & large bowel	Imatinib sensitiv (800 mg/d)
Exon 11		66%	all locations	Imatinib sensitive
Exon 13		1%	all locations	usually secondary mutation resistant to imatinib, responds to sunitinib
Exon 17		<1%	all locations	secondary mutation resistant to imatinib and sunitinib; have been shown to respond to other TKI like regorafenib
***PDGFRA*** **mutations**				
Exon 12		1%	all locations	
Exon 14		<1%	stomach	Imatinib sensitiv
Exon 18 D842V		6%	stomach	Imatinib/sunitinib resistant; good respons to avapritinib
Exon 18 others		1%	all locations	response to imatinib reported
***KIT/PDGFRA* “*wild-type*”**				Limited responses to imatinib Possible response to other TKIs (limited data)
**SDHB IHC+/SDH-competent**	*NF1* mutation (assoc. with RD)	<1%	small bowel	
	*NRAS/HRAS/KRAS* mutations	<1%	all locations (limited data)	
	*BRAF* mutation	1%	most commonly stomach	
	Other rare mutations/fusions		all locations (limited data)	
**SDHB IHC−/SDH-deficient**	*SDHA/B/C/D* mutations (CSS)	2%	stomach	
	Part of the CT *	1%	stomach	
	*SDHA mutation* (young adults)		stomach	
	Sporadic pediatric wt- GIST	1%	stomach	

Legend: CSS: Carney–Stratakis Syndrome; CT: Carney triad; *: most cases show promotor hypermethylation.

**Table 3:** Most common *KIT* and *PDGFRA* mutations found in familial GIST.

*KIT* Mutations	
**Exon 8**	p.D419del
**Exon 9**	p.K509I
**Exon 11**	p.W557R p.V559A p.D579del p.V560G p.V560A p.L576P
**Exon 13**	p.K642E p.N655K
**Exon 17**	p.D820Y p.D820G p.N822Y
***PDGFRA* mutations**	
**Exon 12**	p.V561D p.Y555C
**Exon 14**	p.P653L
**Exon 18**	p.D842Y p.D846V

Adapted from [94].
